# Respiratory distress in a patient with Klinefelter syndrome: a suspicion of COVID-19 hiding severe pulmonary embolism

**DOI:** 10.11604/pamj.supp.2020.37.1.25894

**Published:** 2020-09-18

**Authors:** Elyes Lagha, Rami Tlili, Fares Azaiez, Rym Ben Romdhane, Kawther Bachraoui, Nourelhouda Nouira, Meriam Chaabouni, Youssef Ben Ameur

**Affiliations:** 1 Cardiology Department of Mongi Slim University Hospital, La Marsa, Tunis, Tunisia,; 2 Emergency Department of Mongi Slim University Hospital, La Marsa, Tunis, Tunisia,; 3 Chaabouni Laboratory, Les Jasmins Medical Center, Tunis, Tunisia

**Keywords:** Klinefelter syndrome, COVID-19, pulmonary embolism

## Abstract

Klinefelter syndrome is the most common congenital abnormality causing primary hypogonadism and predisposing to a state of hypercoagulability. We report the case of a 37-year-old man, of Algerian nationality, diagnosed with Klinefelter syndrome admitted to the hospital via the emergency room for acute chest pain and dyspnea. The patient arrived in Tunisia 36 hours ago. On admission, body temperature was 38.2°C, blood pressure, pulse and respiratory rate were 130/70 mmHg, 120/minute and 26/minute, respectively. He had an oxygen saturation of 87% in room air. His electrocardiography revealed a complete right bundle-branch block, chest X-Ray was normal. In front of the clinical presentation and the origin of the patient coming from an endemic country, COVID-19 infection was suspected but ruled out by pharyngeal swabs testing negative by real-time reverse-transcription polymerase chain reaction test and massive pulmonary embolism was diagnosed from his chest computed tomography images. The symptoms improved with anticoagulation treatment.

## Introduction

Klinefelter syndrome (KS) is the most common congenital abnormality causing primary hypogonadism, occurring in approximately one in 500-700 newborns [1]. This syndrome is characterized by the presence of one or more extra X chromosomes [2]. Affected males carry an additional X chromosome (or more), which results in abnormal development of the testis, leading to hypogonadism and infertility. KS has a tendency for hypercoagulability owing to the propensity for hypogonadism caused by hormonal imbalance and genetic inclination [3]. To date, there has been no case report associated with cardio vascular disease in Tunisia. We present a case of pulmonary embolism diagnosed following a suspicion of COVID-19 infection in an adult with known Klinefelter syndrome.

## Patient and observation

**Figure 1:**
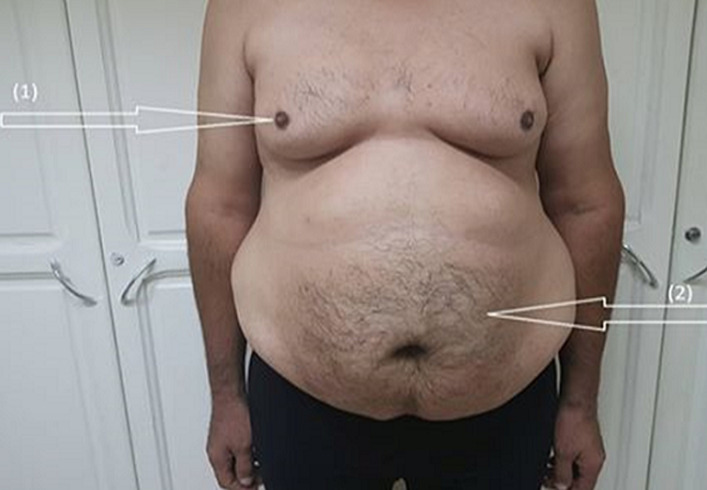
patient's photo visualizing gynecomastia (arrow (1)) and abdominal obesity (arrow (2))

**Figure 2:**
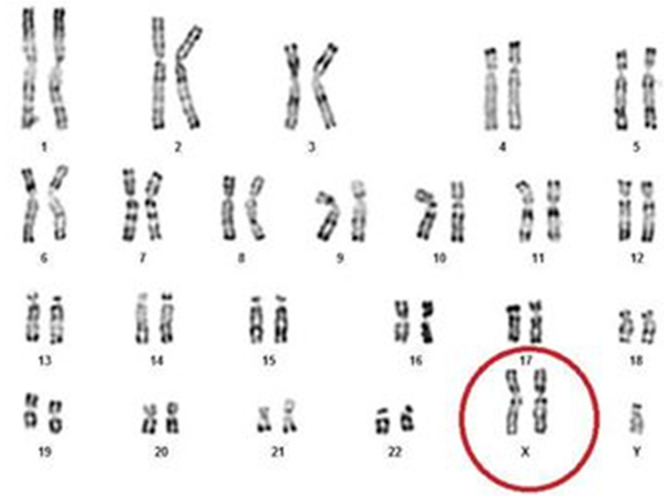
chromosomal analysis revealed a karyotype of 47, XXY, which is a typical finding in Klinefelter syndrome

**Case presentation:** a 37-year-old man, of Algerian nationality, with clinically gynecomastia and abdominal obesity (Figure 1), diagnosed as having KS (47, XXY) during the infertility workup (testicular biopsy and karyotype (Figure 2). This patient, arriving in Tunisia 36 hours ago, was admitted to the hospital at the time of the COVID-19 pandemic for dyspnea and acute chest pain that developed few hours prior to admission. On arrival at the emergency room, his blood pressure, pulse and respiratory rate were 130/70 mmHg, 120/minute and 26/minute, respectively. His body temperature was 38.2°C and he was clearly conscious. The auscultation showed rapid pulse and no pulmonary rales. The patient was cyanotic with an oxygen saturation of 87% in room air. COVID-19 infection was suspected in the first place. Clinic probability of pulmonary embolism has been classified as intermediate (WELLS SCORE calculated= 4.5).

**Figure 3:**
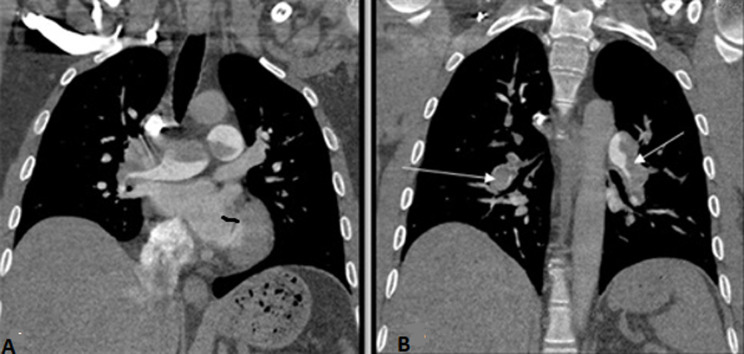
coronal reconstructions of a pulmonary angiogram, in a mediastinal window, showing the endoluminal defect of the right and left pulmonary arteries (A) extended to all the segmental branches (B)

**Investigations:** the electrocardiography revealed a complete right bundle-branch block. Chest X ray was normal. Blood tests revealed white blood cells: 6980 U/L, hemoglobin: 13 g/dL and platelet: 267000 U/L. Serobiochemical studies showed blood urea nitrogen: 4.5 mmol/l, creatinine: 77 μmol/L. C reactive protein 40 mg/mL, creatine kinase-MB, Brain Natriuretic Peptide (BNP) and pro-BNP were all negative, while the level of troponin was measured to be high at 1669 ng/ml. D-dimers levels were measured and the test came positive with the high value of 2560 μg/L. Therefore, Specific RT-PCR on a nasopharyngeal swab was performed and a chest computed tomography (CT) was carried out showing no signs of SARS-CoV-2 infection but revealing a massive bilateral proximal pulmonary embolism (PE) (Figure 3). A transthoracic echocardiography showed dilated right ventricle with preserved function, pulmonary artery hypertension and a concentrically enlarged left ventricular. After 48 hours the result of the RT-PCR was negative and the COVID-19 was ruled out.

**Treatment and outcome:** simplified Pulmonary Embolism Severity Index (sPESI) score calculated=1 (high level of troponin and tachycardia 120/minute) so it was a PE with an intermediate risk of mortality. The patient was hospitalized in a cardiological intensive care unit, anticoagulant therapy was initiated and the patient has shown considerable improvements.

## Discussion

The clinical presentation of our patient with a respiratory distress and fever 36 hours after a trip from Algeria to Tunisia prompted caregivers to evoke a SARS-CoV-2 respiratory infection. At that time Algeria was the most affected African country and any patient coming to Tunisia from any endemic country was classified as a COVID-19 possible case [4]. During this pandemic, the suspicion of COVID-19 in a patient could mislead the doctor by considering a single diagnosis and not think of bringing up other more obvious diagnoses that could put the patient's vital prognosis at risk immediately. In our case the final diagnosis was massive pulmonary embolism in a patient with klinefelter syndrome. There is increased incidence of venous thromboembolism (VTE) in patients affected by KS. According to Campbell *et al*., the incidence of deep vein thrombosis was about 22 cases per 10000 in known KS patients aged 30 to 70 years compared to 4 cases per 10000 in the general population [5]. These data were confirmed in the Danish cohort with a relative risk of 5.29 for deep vein thrombosis and 3.60 for PE [6]. The increased thromboembolic risk is explained by hypofibrinolysis linked to androgen deficiency [7]. Overweight could also play a role. Through such pathogenesis, patients with KS would have a higher risk of developing myocardial infarction and VTE. However, it would be difficult to explain severe thromboses with hormonal imbalance by way of hypo-androgenism alone, and one or more inherited thrombophilia {e.g., factor V Leiden and prothrombin (factor II) G20210A mutations} may be associated with it. Therefore, patients with KS with a past medical or family history of VTE should undergo an endocrinologic test, as well as additional assessment of innate or acquired thrombophilia [8]. In the future, it seems that more studies on understanding of pathogenesis of VTE in cases of KS are necessary. The consideration is that a long-term oral anticoagulation therapy is necessary for the treatment of thromboembolic complications, prophylactic anticoagulation, in these patients, may be necessary in some situations where the risk of recurrence is very high.

## Conclusion

The novel coronavirus is a serious threat but the focus should not be solely diverted to the COVID-19. During this pandemic vital diagnosis such as pulmonary embolism should not be missed as it could cost patient´s lives, especially with pathological conditions like KS predisposing to thromboembolic complications.
